# Repetition enhances the effects of activated long-term memory

**DOI:** 10.1177/17470218221095755

**Published:** 2022-05-25

**Authors:** Lindsay Plater, Sandra Nyman, Samantha Joubran, Naseem Al-Aidroos

**Affiliations:** Department of Psychology, University of Guelph, Guelph, Ontario, Canada

**Keywords:** Visual long-term memory, activated long-term memory, intrusion effect, attention

## Abstract

Recent research indicates that visual long-term memory (vLTM) representations directly interface with perception and guide attention. This may be accomplished through a state known as activated LTM, however, little is known about the nature of activated LTM. Is it possible to enhance the attentional effects of these activated representations? And furthermore, is activated LTM discrete (i.e., a representation is either active or not active, but only active representations interact with perception) or continuous (i.e., there are different levels within the active state that all interact with perception)? To answer these questions, in the present study, we measured intrusion effects during a modified Sternberg task. Participants saw two lists of three complex visual objects, were cued that only one list was relevant for the current trial (the other list was, thus, irrelevant), and then their memory for the cued list was probed. Critically, half of the trials contained repeat objects (shown 10 times each), and half of the trials contained non-repeat objects (shown only once each). Results indicated that repetition enhanced activated LTM, as the intrusion effect (i.e., longer reaction times to irrelevant list objects than novel objects) was larger for repeat trials compared with non-repeat trials. These initial findings provide preliminary support that LTM activation is continuous, as the intrusion effect was not the same size for repeat and non-repeat trials. We conclude that researchers should repeat stimuli to increase the size of their effects and enhance how LTM representations interact with perception.

In an attempt to understand how long-term memory (LTM) guides attention, visual attention researchers have started to look at the role of memory state (Plater et al., 2020; Wolfe, 2012) described by embedded-processes models of memory (Cowan, 1988; Oberauer, 2002; Oberauer & Hein, 2012). For example, it has recently been demonstrated that visual LTM (vLTM) representations can interact with perception to determine the types of stimuli that reflexively capture attention (Fan & Turk-Browne, 2016; Fukuda & Vogel, 2019; Wolfe, 2012). Drawing from embedded-processes models, one possible explanation is that LTM representations guide attention through a state known as *activated LTM* (Cunningham & Wolfe, 2014; Oberauer, 2001, 2002; although see Plater et al., 2020 for an example where this is not the case). Because activated LTM representations are more accessible for ongoing cognitive processing than non-activated LTM representations (Oberauer, 2002), these representations are well-positioned to influence observers’ attentional goals and the selection of visual perceptual representations. While this account holds promise for advancing our understanding of the relationship between LTM and visual attention, before testing the account it would help to have a better understanding of the general characteristics of activated LTM. Thus, in the present study, we examined the role of repetition: When the representation of a visual stimulus in LTM is repeatedly placed in the activated state, does the activated representation have a greater capacity to influence other aspects of cognition? Put differently, does repetition enhance the effects of activated LTM?

## Assessing whether a LTM representation is in the active state

The embedded-processes model of memory often includes four distinct memory states: LTM, activated LTM, the region of direct access, and the focus of attention. In the present research, we use Oberauer’s definition for activated LTM: that these representations are a subset of LTM available for ongoing cognitive processes (Oberauer, 2002, 2009; Oberauer & Hein, 2012). As a point of clarification, however, the phrasing “activated LTM” refers specifically to the memory *state*, and does not necessarily relate to other types of memory “activation,” such as spreading activation (i.e., priming) or neurological activation. Indeed, the term “activated LTM” is perhaps a misnomer, as Lewis-Peacock et al. (2012) have shown using fMRI classifier decoding that activated LTM representations are maintained without ongoing neural activity.

As a first step in assessing the effects of repetition on activated LTM, we need a way to determine whether an LTM representation is in the active state. While researchers have suggested that both visual working memory (Eimer & Kiss, 2010; Olivers, 2009; Olivers et al., 2011; although see Beck et al., 2012) and LTM (Cowan, 1988, 2008; Oberauer, 2001, 2002) have active and non-active states, there appears to only be one current methodology for assessing activated LTM: the *intrusion effect* during a modified Sternberg task (Oberauer, 2001; Sternberg, 1966). Oberauer (2001) had participants memorise two shortlists of words and then indicated to participants via a cue which list was relevant for the current trial. The other list was, therefore, irrelevant (note that this is similar to—though possibly distinct from—the directed forgetting literature, where participants are explicitly instructed to forget a subset of items. See: MacLeod, 1998). The idea is that, following the cue, the irrelevant list items should be removed from working memory but may nevertheless be represented in activated LTM and influence task performance. This is what he found. When the memory of the relevant list was probed, irrelevant list items produced an intrusion effect. That is, it took participants longer to reject words from the irrelevant list than novel probes, suggesting irrelevant stimuli were still represented in activated LTM. We have recently shown that this effect holds when having participants remember visual objects rather than words, suggesting that this task can also be used to assess the activated state in vLTM (Plater et al., 2020). In summary, this modified Sternberg task can be used to assess whether an LTM representation is in the active state. Moreover, this task affords a prediction: If repetition can enhance the effects of activated LTM, then stimuli that are repeatedly stored in activated LTM should produce larger intrusion effects than stimuli that are stored in activated LTM only once.

## Is repetition likely to enhance the effects of activated LTM?

While it has long been known that repetition can enhance LTM retention generally (Ebbinghaus, 1913; Hebb, 1961), no studies to date appear to have tested for the effects of repetition on activated LTM. Is the activated LTM state discrete (i.e., a representation is either activated or not), or is it continuous? If activated LTM is discrete, then there should be no effect of repetition (i.e., no change in the intrusion effect), as the representation is either activated or not. If activated LTM is continuous, however, then repetition could alter the magnitude of the intrusion effect. There is some evidence that the activated state is continuous, which would potentially allow for enhancements from repetition (Cowan, 1988; Oberauer, 2001, 2002; Oberauer & Hein, 2012). In particular, using a modified Sternberg task, Oberauer (2001) found that intrusion effects decreased slightly as the time increased between memory array and probe. That is, the activated memory state appeared to decay slightly over time. This finding could indicate that activated LTM is a continuous state, with activation decreasing gradually over time. Because intrusion effects were calculated across many trials, however, it is difficult to disentangle whether decay was gradual or occurred suddenly but at different points in time for different representations (cf., Zhang & Luck, 2009). Nevertheless, the decay of the intrusion effect hints at the potential for repetition to enhance the effects of activated LTM.

## The present study

In the present study, we tested whether repetition affects activated LTM using a modified version of Oberauer’s (2001) modified Sternberg task. Given the relevance of this question for ongoing research on interactions between LTM and visual attention, we focus here specifically on activated vLTM. Participants saw two lists that each contained three complex visual objects on every trial. Participants were cued which list was relevant (the other list was, thus, irrelevant), and their memory for the objects from the relevant list was then probed. Similar to both Oberauer (2001) and Plater et al. (2020), the memory probe could be one of the following: a relevant list object, an irrelevant list object, or a novel object. New for this study, we assessed the effect of repetition on the intrusion effect. Half of the trials contained complex visual objects that were presented only one time each (non-repeat objects), and the other half of the trials contained objects that were presented multiple times each (repeat objects). Our primary interest for this study was to assess, overall, if repetition can increase the intrusion effect, rather than to assess how the intrusion effect changes with each individual repetition. Accordingly, for trials with repeat objects, we presented a small number of objects many times (10 repetitions) rather than many objects a small number of times. If the effects of activated LTM can be enhanced through repetition, then we should observe larger intrusion effects by repeat objects than non-repeat objects.

## Experiment 1

### Method

#### Participants

An *a priori* power analysis was conducted using RStudio version 1.3.1093 (RStudioTeam, 2020) to determine the minimum sample size necessary to detect the critical effect for both experiments. We used the following effect sizes for the intrusion effect (i.e., longer reaction times to irrelevant than novel probes, with complex visual objects shown once) from Plater et al. (2020): *d* = 1.06 in Experiment 1, *d* = 0.83 in Experiment 2, and *d* = 2.52 in Experiment 3. Alpha was set to 0.05, and power was set to 0.80, resulting in a required sample of 3 to 11 participants required to find the basic intrusion effect. Due to the additional complexity of the current analysis (i.e., an analysis of variance [ANOVA] to determine if repetition increases the intrusion effect), a larger sample than Plater et al. (2020) Experiment 1 (*n* = 50) was used for the current experiments.

Sixty-five undergraduate students (mean age 18.8 years, 56 women and 9 men) from the University of Guelph participated for partial course credit. All participants reported having normal colour vision and normal, or corrected-to-normal, visual acuity. Five participants were excluded from the analyses for failing to follow task instructions (two for not finishing the experiment, and three for pressing the wrong buttons or no buttons), and three participants were excluded from the analyses for error rates of 40% or higher on the working memory task (as per: Plater et al., 2020). Written informed consent was obtained from each participant, and experimental protocols were approved by the University of Guelph research ethics board (approval number 17-05-003).

#### Apparatus, stimuli, and procedure

The experiment was conducted in a laboratory on a desktop computer with a 1,280 × 1,024 resolution, 75 Hz CRT display. Responses were made using an Xbox 360 controller, which was connected to the computer through USB. Participants used a head and chin rest to keep their gaze distance constant at 52 cm from the computer screen for the duration of the experiment.

**Figure 1.**
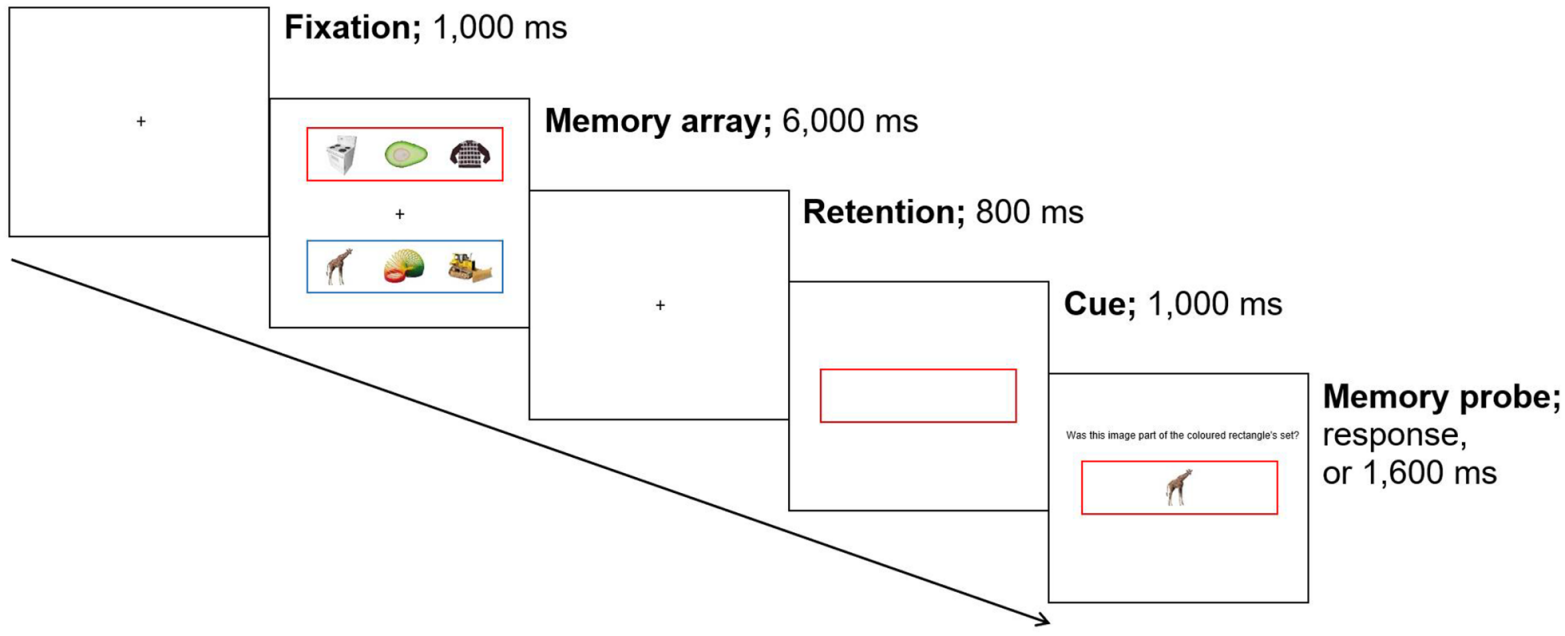
Example trial sequence of the working memory task; participants must report whether the memory probe was present in the cued (relevant) list on the current trial. This is a depiction of an irrelevant trial, as the memory probe was part of the uncued (irrelevant) list.

Participants completed 240 trials of a modified version of Oberauer’s (2001) modified Sternberg task (see Figure 1), with breaks every 24 trials. Each trial started with a white fixation screen that contained a black central fixation cross that measured 1 × 1°, presented for 1,000 ms. The fixation screen was followed by a memory array, which displayed two lists that each contained three complex visual objects for 6,000 ms; one list was presented in a red rectangular frame 10° above the centre of the screen, and the other list was presented in a blue rectangular frame 10° below the centre of the screen. Both frames measured 5° high, 30° wide, and had lines that were 0.04° thick. Each object subtended 3.44 × 3.44° and appeared in one of three possible locations within the rectangular frame: 10° to the left of centre, central, or 10° to the right of the centre. The memory array was followed by an 800 ms memory delay. Next, on the cue screen, the fixation cross was replaced with a central rectangular frame (5° high, 30° wide, lines 0.04° thick); this 100% valid cue was randomly presented in red or blue and indicated to participants which memory list would be probed at the end of the trial (i.e., the *relevant list*) and, consequently, which list would not be probed (i.e., the *irrelevant list*). The cue was presented for 1,000 ms before the memory probe was added to the display. The 3 × 3° memory probe object was presented in the centre of the rectangular cue and could be: a relevant list object (*relevant probe*, 50% of trials, requires a “yes” response), an irrelevant list object (*irrelevant probe*, 25% of trials, requires a “no” response), or a novel object (*novel probe*, 25% of trials, requires a “no” response). Participants had 1,600 ms to respond to whether the memory probe had been presented in the relevant list on the current trial; if the participant was incorrect, or failed to respond in time, a 500-Hz error tone sounded for 50 ms.

All object images for this task were presented on a white background and were selected from Brady et al. (2008). Objects spanned many categories, including food, animals, general household objects, tools, clothing, appliances, vehicles, and buildings. Six unique objects were presented in the memory array on every trial and were either all repeat objects or all non-repeat objects. There were 72 *repeat objects* presented 10 times each. Throughout the experiment, each repeat object was randomly presented once as a memory array object before the repeat objects were shuffled and randomly presented again. These repeat objects were randomly selected without replacement from a pool of 125 object images for each participant. We chose to use a relatively large number of repetitions of the repeat objects to increase the likelihood of finding a repetition effect if one exists. While this design does not afford enough power to assess how the intrusion effect changes across each repetition, such an analysis would likely not be possible even if we used fewer repetitions of more repeat objects. That is, with a 30–35% error rate (Plater et al., 2020), we only expect about 20 trials per participant in each of the critical irrelevant probe conditions. As the probe object was randomly decided on each trial, the repeat objects could appear as a relevant probe and/or as an irrelevant probe, or they could never be used as a probe. There were 720 *non-repeat objects.* Each non-repeat object was presented as a memory array object once throughout the experiment. These non-repeat objects were randomly selected without replacement from a pool of 2,152 object images for each participant. Sixty novel objects were randomly selected without replacement from the same pool of 2,152 object images for each participant. Each novel object was presented as a memory probe once throughout the experiment. Probe type (relevant, irrelevant, or novel) and the trial’s repeat status (repeat or non-repeat) were fully crossed and randomised throughout the experiment for each participant.

### Results

Reaction time (RT) data were trimmed of outliers. For each participant, trials with an RT >2.5 *SD* from the condition mean were removed (1.49% of trials). Incorrect response trials (20.0%) and timeout trials (3.91%) were also excluded from RT analyses.

**Figure 2.**
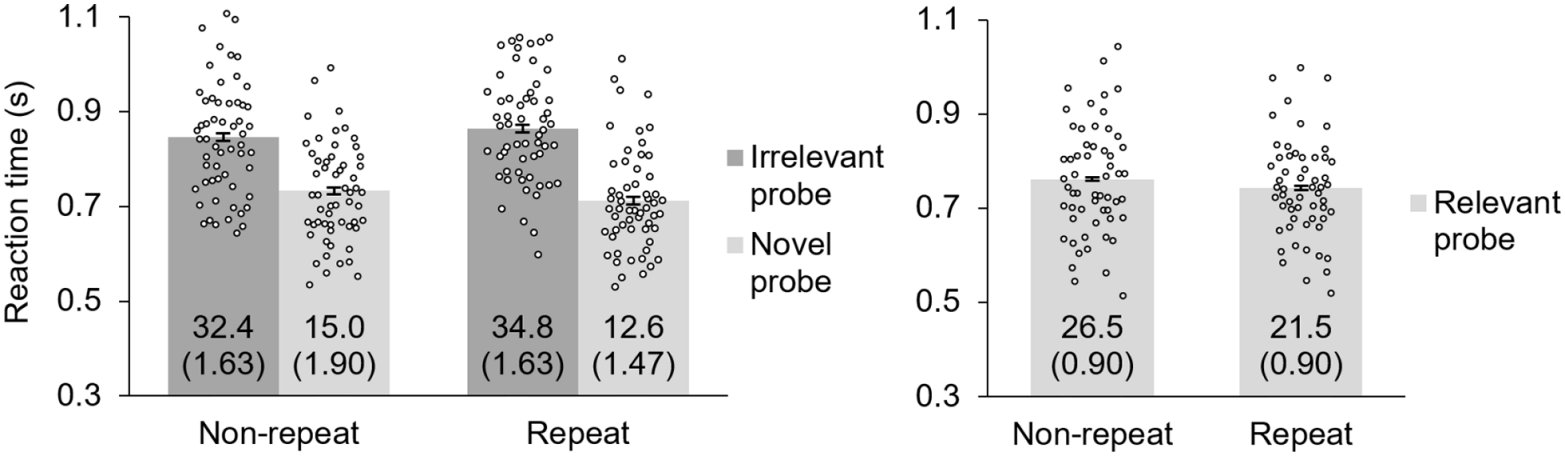
Reaction time data for working memory task of Experiment 1. Irrelevant and novel probes (left graph) require a “no” response and are of primary interest for the present study as they are used to calculate the intrusion effect. Participants exhibited an intrusion effect (longer reaction times to irrelevant than novel probes) for both repeat and non-repeat objects; this effect was larger for repeat objects. Relevant probes (right graph) require a “yes” response and are not used to calculate the intrusion effect. Error bars in all figures are corrected (Morey, 2008) within-subject standard errors (Cousineau, 2005). Numbers inside the bars in all figures indicate the error percentage (and corrected within-subject standard errors) for each condition. Dots on each bar in all figures indicate the average reaction time for each participant for each condition.

RT data for trials with relevant probes are plotted in Figure 2 on the right (note that these trials require a “yes” response, and are not pertinent for measuring the intrusion effect). A paired-samples *t* test indicated faster RTs for the repeat relevant probes than the non-repeat relevant probes: *t*(56) = 3.03, *p* < .001, *d* = 0.40.

RT data for trials with irrelevant probes and novel probes are plotted in Figure 2 on the left (note that these trials require a “no” response). A 2 (probe type: irrelevant vs. novel) × 2 (repeat status: repeat vs. non-repeat) within-subjects ANOVA was conducted to assess for differences in RTs across conditions. The interaction of probe type and repeat status was significant, *F*(1, 56) = 7.19, *p* = .010, η_p_^2^ = .114, which demonstrates that the size of the intrusion effect differed when the probe was a repeat object than when it was a non-repeat object. In addition, the main effect of probe type was significant, *F*(1, 56) = 205, *p* < .001, η_p_^2^ = .785, and the main effect of repeat status was not significant, *F*(1, 56) = 0.04, *p* = .853, η_p_^2^ = .001.

**Figure 3.**
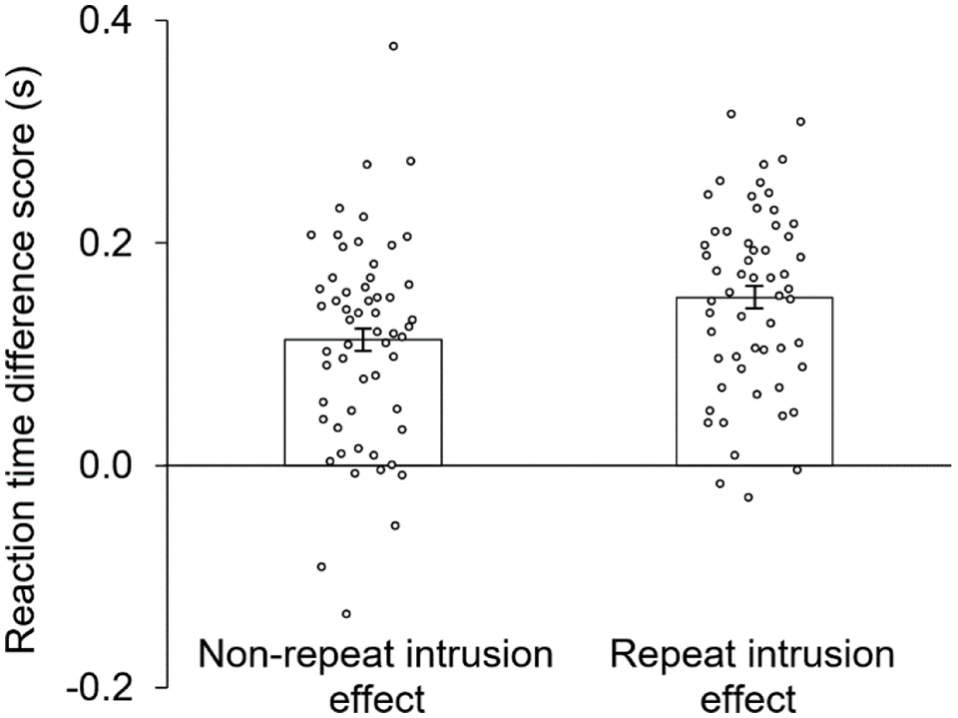
The intrusion effect for Experiment 1’s non-repeat and repeat conditions was calculated as the difference between irrelevant probes and novel probes. Objects that were repeated produced a larger intrusion effect than objects that were not repeated.

To investigate these differences further, two planned paired-samples *t* tests were conducted to verify that both repeat and non-repeat objects produced an intrusion effect; these tests revealed that irrelevant objects produced longer RTs than novel objects for both repeat trials, *t*(56) = 13.8, *p* < .001, *d* = 1.83, and non-repeat trials, *t*(56) = 9.15, *p* < .001, *d* = 1.21, indicating that the vLTM representations of irrelevant list objects from both trial types were successfully in the active state. To better visualise the enhancement of the intrusion effect produced by repetition from Figure 2, a difference score (irrelevant probe—novel probe) was calculated for both the repeat and the non-repeat conditions for each participant; the average of these difference scores can be seen in Figure 3. The effect of repetition on the intrusion effect was significant (see two-way interaction earlier), and associated with moderate effect size, *d* = 0.36. To quantify this effect, repeating an object 10 times increased the size of the intrusion effect by 34.0% (i.e., 38.4 ms, plus or minus 28.1 ms) compared with showing an object only once.

Error rates are indicated in Figure 2 in numerals. To assess for potential speed-accuracy trade-offs, the same 2 × 2 ANOVA and *t* tests were conducted on error rates. The relevant probe *t-*test revealed shorter RTs for repeat than non-repeat probes, *t*(56) = 3.95, *p* < .001, *d* = 0.52. The ANOVA revealed a significant interaction between probe type and repeat status, *F*(1, 56) = 4.58, *p* = .037, η_p_^2^ = .076, a significant main effect of probe type, *F*(1, 56) = 64.0, *p* < .001, η_p_^2^ = .533, and a non-significant main effect of repeat status, *F* < 1. In addition, the paired-samples *t* tests revealed significantly more errors for the irrelevant probe condition than for the novel probe condition for both non-repeat trials, *t*(56) = 6.54, *p* < .001, *d* = 0.87, and repeat trials, *t*(56) = 8.00, *p* < .001, *d* = 1.06. Importantly, for all instances of significant differences in error rates, participants made more errors for the slower condition; thus, it is unlikely that the observed differences in RTs were due to speed/accuracy trade-offs.

## Experiment 2

There was a potential confound in Experiment 1: Repeat objects were randomly selected from a different subset of object images than non-repeat and novel objects. While all object images were originally from the same set (Brady et al., 2008), there could have been minor differences in the object images used for each condition that accounts for the larger intrusion effect for repeat versus non-repeat stimuli, such as greater memorability (Rust & Mehrpour, 2020; Standing, 1973), a specific object category (Brady et al., 2008), or object shape or colour (Reppa et al., 2020). To address this issue, Experiment 2 followed the same methods as Experiment 1, but all object images were randomly selected for each participant from the same set of images. We found a comparable increase in the magnitude of the intrusion effect across the two experiments. While the experimental design was adjusted slightly for online data collection (i.e., face-to-face research was not possible for Experiment 2), we nevertheless conclude that the objects themselves were unlikely to determine the size of the intrusion effect for repeat and non-repeat objects in Experiment 1.

### Method

#### Participants

The same *a priori* power analysis from Experiment 1 was used to determine the sample size for Experiment 2. While a sample size of 65 was sufficient to find the intrusion effect in Experiment 1, a larger sample was tested in Experiment 2 due to the switch to online data collection to account for: (a) possible higher attrition and/or exclusion rates and (b) potentially more variable data.

Seventy-nine participants (mean age 27.3 years, 40 women and 39 men) from the Prolific.co (Prolific, 2014) online participant pool participated in Experiment 2 for a £3 payment. The experiment was created using the PsychoPy Builder (v2020.1.3) with some code components (Peirce et al., 2019) and output to a PsychoJS experiment that was housed on Pavlovia.org (Pavlovia, 2018). Importantly, online studies have been shown to have accurate timing (Bridges et al., 2020), and Prolific has been shown to replicate known psychological effects (Palan & Schitter, 2018; Peer et al., 2017).

All participants reported having a normal colour vision and normal, or corrected-to-normal, visual acuity. All participants were 18 years of age or older. Ten participants were excluded from the analyses for error rates of 40% or higher on the working memory task, and two participants were excluded from the analyses for having no data in one of the four conditions required for the analyses. Written informed consent was obtained from each participant, and experimental protocols were approved by the University of Guelph research ethics board (approval number 20-06-039).

#### Apparatus, stimuli, and procedure

Relative to Experiment 1, the following changes were made to Experiment 2. Participants only completed 120 trials of the task (see Figure 1), rather than 240. As such, the number of repeat objects was reduced from 72 to 36, and non-repeat objects from 720 to 360. This decrease helped reduce the number of object images and, accordingly, how long it took participants to download the experiment to their device. Moreover, in our experience, shorter experiments (i.e., less than 30 min) are more effective for online studies. Importantly, as in Experiment 1, repeat objects were still shown 10 times each, and non-repeat objects were still shown only 1 time each.

As participants completed the experiment on their personal laptop or desktop computer, we were not able to control the size of stimuli as precisely as in Experiment 1. Stimulus sizes were all specified as a percentage of the height of the participants’ screen. For example, the fixation cross was 1.4% of the height of each participant’s screen tall, and 1.4% of the height of each participant’s screen wide. Thus, stimuli appeared in different sizes to different participants. To better appreciate how stimuli appeared to each participant, we asked them to self-report the height of their computer screen in inches and the distance from their screen to their eyes. The average screen height was 10.2 inches (*SD* = 3.06 inches), and the average distance was 19.6 inches (*SD* = 6.49 inches). This means that the average height of participants’ screens subtended 31.4° (*SD* = 12.2°). Thus, on average, the fixation point was 0.4° in width and height (i.e., 1.4% of 31.4°).

Stimulus sizes for Experiment 2, specified as percentages of screen height, were as follows. The rectangular frames were 10% tall and 60% wide (presented 20% above or below fixation, or centrally for the cue rectangle). Object images were 8% in width and height and were presented centred vertically within a rectangular frame, and 20% to the left or right of fixation or centred horizontally. There was no error tone for Experiment 2, but participants were given visual feedback on each trial. The word “Correct.” or “Incorrect.” (2.5% in height, in black Arial font) was presented for 500 ms before the start of the next trial.

The critical change in Experiment 2 was that all object images (36 repeat objects, 360 non-repeat objects, 30 novel objects) were randomly selected for each participant from a single set of 426 object images. This allows us to determine whether the effects found in Experiment 1 were due to the repeat and non-repeat object images being selected from different sets of images, or whether repeated presentation of object images does indeed affect the magnitude of the intrusion effect.

### Results

RT data were trimmed out of outliers. For each participant, trials with an RT greater than 2.5 standard deviations from the condition mean were removed (1.12% of trials). Incorrect response trials (19.3%) and timeout trials (4.18%) were also excluded from RT analyses.

**Figure 4.**
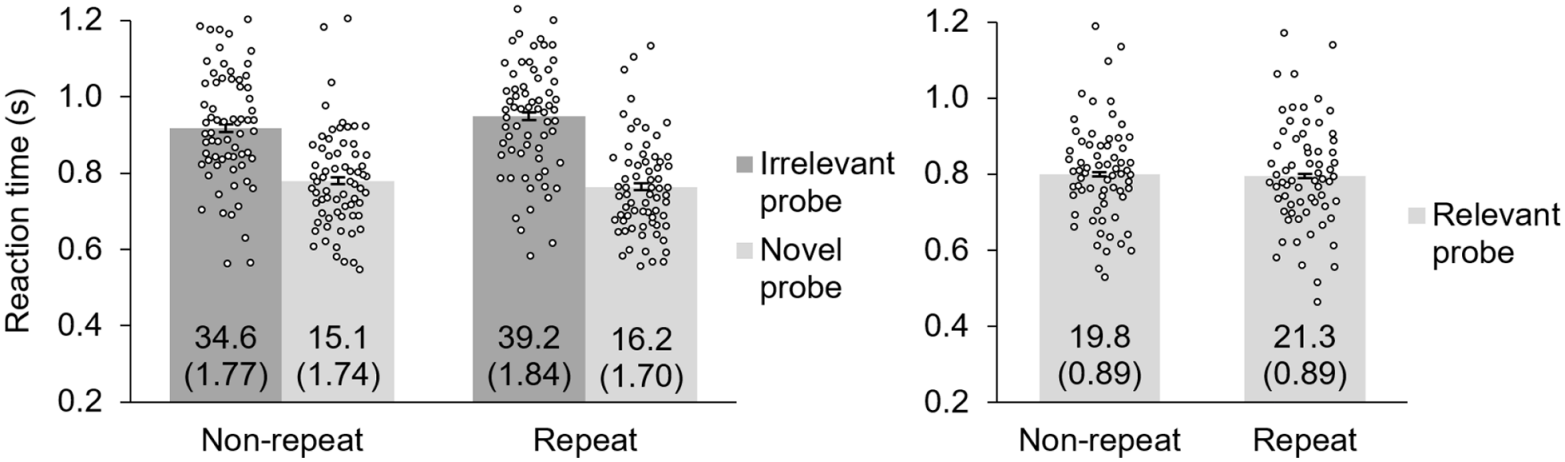
Reaction time data for working memory task of Experiment 2. Numbers inside the bars indicate the error percentage for each condition. Irrelevant and novel probes (left graph) require a “no” response and are of primary interest for the present study as they are used to calculate the intrusion effect. Participants exhibited an intrusion effect (longer reaction times to irrelevant than novel probes) for both repeat and non-repeat objects, but this effect was larger for repeat objects. Relevant probes (right graph) require a “yes” response and are not used to calculate the intrusion effect.

RT data for trials with relevant probes are plotted in Figure 4 on the right.

The paired-samples *t* test for repeat relevant probes versus non-repeat relevant probes was not significant: *t*(66) = 0.75, *p* = .46, *d* = 0.09.

RT data for trials with irrelevant probes and novel probes are plotted in Figure 4 on the left. Similar to Experiment 1, a 2 (probe type: irrelevant vs. novel) × 2 (repeat status: repeat vs. non-repeat) within-subjects ANOVA was conducted to assess for differences in RTs across conditions. These results replicated the Experiment 1 results. The interaction of probe type and repeat status was significant, *F*(1, 66) = 8.05, *p* = .006, η_p_^2^ = .109, which demonstrates that the size of the intrusion effect differed when the probe was a repeat object than when it was a non-repeat object. The main effect of probe type was significant, *F*(1, 66) = 184, *p* < .001, η_p_^2^ = .736, and the main effect of repeat status was not significant, *F*(1, 66) = 1.24, *p* = .269, η_p_^2^ = .019.

**Figure 5.**
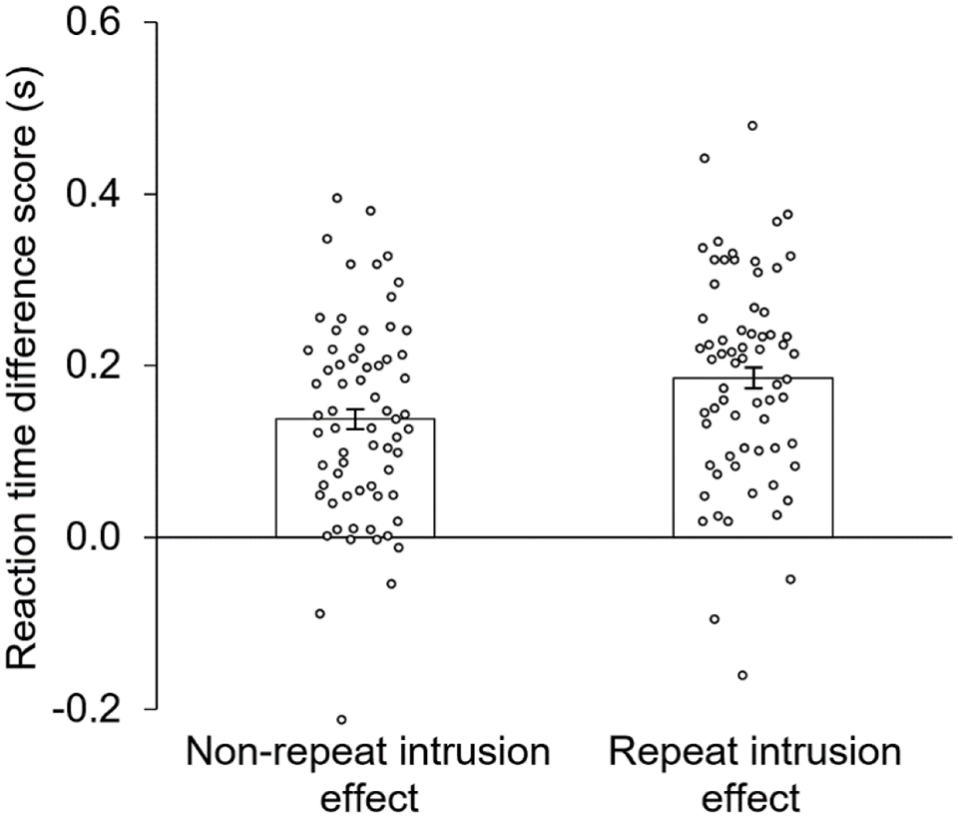
The intrusion effect for Experiment 2’s non-repeat and repeat conditions was calculated as the difference between irrelevant probes and novel probes. Objects that were repeated produced a larger intrusion effect than objects that were not repeated.

To investigate these differences further, the same two planned paired-samples *t-*tests as conducted in Experiment 1 were conducted to verify that both repeat and non-repeat objects produced an intrusion effect. These results replicated Experiment 1’s results and revealed that irrelevant objects produced longer RTs than novel objects for both repeat trials, *t*(66) = 12.4, *p* < .001, *d* = 1.51, and non-repeat trials, *t*(66) = 9.67, *p* < .001, *d* = 1.18, indicating that the vLTM representations of irrelevant list objects from both trial types were successfully in the active state. In addition, the same difference scores as Experiment 1 were calculated for Experiment 2 (see Figure 5). Again, the effect of repetition on the intrusion effect was significant (see two-way interaction above), and was associated with moderate effect size, *d* = 0.35. Here, repeating an object 10 times increased the size of the intrusion effect by 34.9% (i.e., 48.1 ms, plus or minus 33.2 ms) compared with showing an object only once. As all objects in Experiment 2 were randomly selected from the same pool of object images, the differences found here and in Experiment 1 were not caused by differences in the source of the object images. Rather, repeating memory array objects increases the intrusion effect.

Error rates are indicated in Figure 4 in numerals. To assess for potential speed-accuracy trade-offs, the same 2 × 2 ANOVA and *t* tests were conducted on error rates. The relevant probe *t-*test was not significant, *t*(66) = 1.22, *p* = .23, *d* = 0.15. The ANOVA revealed a non-significant interaction between probe type and repeat status, *F*(1, 66) = 3.24, *p* = .077, η_p_^2^ = .047, a significant main effect of probe type, *F*(1, 66) = 61.2, *p* < .001, η_p_^2^ = .481, and a significant main effect of repeat status, *F*(1, 66) = 8.03, *p* = .006, η_p_^2^ = .108. In addition, the paired-samples *t* tests revealed significantly more errors for the irrelevant probe condition than for the novel probe condition for both non-repeat trials, *t*(66) = 6.81, *p* < .001, *d* = 0.83, and repeat trials, *t*(66) = 7.93, *p* < .001, *d* = 0.97. Importantly, for all instances of significant differences in error rates, participants made more errors for the slower condition; thus, it is unlikely that the observed differences in RTs were due to speed/accuracy trade-offs.

## Discussion

In the present study, we assessed the effects of repetition on activated LTM. We used a modified Sternberg task to induce participants to represent complex visual objects in activated vLTM and found that objects that were presented 10 times produced an intrusion effect that was 34% (Experiment 1) or 35% (Experiment 2) larger than the intrusion effect for objects that were presented only once. Thus, repetition enhances the effects of activated LTM. In addition, this finding adds to the evidence that the activated state in LTM is continuous rather than discrete. That is, while both repeat and non-repeat objects were represented in the activated state, one interpretation of the present findings is that repeat object representations were more activated.

The critical finding from this research is that repeating stimuli can enhance the effects of activated LTM. While it may seem obvious that repeating stimuli would lead to larger memory-related effects (in this case, a greater intrusion effect), this is not always the case. When observers search their environment for any of a set of visual objects, they use vLTM representations to specify an attentional set that causes only those objects to capture attention (Giammarco et al., 2016). We recently tested whether this control over attentional capture is influenced by how often participants have observed and attended to these objects and found no effect (Giammarco et al., 2021). Thus, repetition does not enhance our ability to use LTM representations to control attentional capture. Interestingly, we have also shown that this ability to control attentional capture is not accomplished by representing the searched-for objects in activated LTM (Plater et al., 2020). Accordingly, these studies converge with the present results: Repetition enhances effects related to activated LTM such as the intrusion effect, and not effects unrelated to activated LTM such as the control of attentional capture. More importantly, these studies highlight how a better understanding of the effects of repetition can inform the investigation of potential relationships between activated LTM and attention in general. At the very least, given that repetition can specifically bolster the effects of *activated* LTM, researchers investigating the interaction of attention and activated LTM should repeat their stimuli to increase the size of the effect of interest.

### What is the intrusion effect?

The intrusion effect is a difference score, calculated as the difference in RTs to irrelevant probes versus novel probes. When probing memory for the cued list, why do the uncued, irrelevant list probes produce longer reaction times than novel probes? While the magnitude of the intrusion effect can be influenced by changes to the irrelevant probe RT, the novel probe RT, or both, Oberauer (2002) suggests the intrusion effect is likely attributed to a familiarity signal elicited by irrelevant list items. That is, retrieving the irrelevant list item from LTM elicits a rapid sense of familiarity that the item has been seen before. This familiarity signal has to be overridden by slower recollective memory processes that indicate the item is, in fact, from the irrelevant list (Moscovitch, 2008; Oberauer, 2002). Importantly, the novel probes do not elicit the same familiarity signal, so participants make their response to novel probes more quickly and with fewer errors. In the present experiment, novel probes were shown only one time each (note that “repeat” and “non-repeat” trials refer to the memory array objects) and were randomly chosen for each participant from the same pool of objects. Thus, there should be no difference in the familiarity signal for novel objects.

Similarly, when probing memory for the cued list, why do the repeat irrelevant list probes elicit a larger intrusion effect than non-repeat irrelevant list probes? Given Oberauer’s (2002) familiarity explanation of the intrusion effect, it is likely to be the case that repeating objects multiple times throughout the experiment strengthens the familiarity signal of these repeat objects, thus leading to longer reaction times, as participants have to overcome this heightened sense of familiarity to correctly reject the irrelevant list items. Thus, the larger intrusion effect for repeat objects compared with non-repeat objects is likely a result of this increased familiarity signal that is a by-product of greater activation for the repeat versus non-repeat objects. There are other possibilities that could explain why repeat objects produced longer RTs than non-repeat objects, such as episodic confusion (i.e., “Wait, was this item in the relevant list, or the irrelevant list?”) or interference based on previous experience with an item. However, we would expect the repeat objects shown as relevant probes to show similar episodic confusion/interference, thus also producing longer RTs for repeat than non-repeat probes. Instead, we found faster RTs for repeat than non-repeat relevant probes (although only significantly faster in Experiment 1).

### The consequence of repeating stimuli

It is well-documented that repeating stimuli during an experiment may change how we process those stimuli. As just three examples: repeating stimuli has been shown to improve memory accuracy for those stimuli (Hebb, 1961; Oberauer et al., 2015), repeating stimuli has been shown to lead to faster responses (i.e., repetition priming) to those stimuli (Desimone, 1996; Grill-Spector et al., 2006; Henson & Rugg, 2003; Schacter & Buckner, 1998), and repeating stimuli has been shown to decrease the hemodynamic response (i.e., repetition suppression) to those stimuli (Barron et al., 2016). The results of the present research, as well as other research using Sternberg tasks (Oberauer, 2001, 2005; Plater et al., 2020), suggest that RTs may be inflated when responding to stimuli that have activated LTM representations. Furthermore, our results suggest that RTs may be inflated to an even greater extent if stimuli are presented multiple times. Researchers should be aware of this potential for increased RTs, as repeating stimuli may have the same effect for other kinds of tasks as well, including possibly: hybrid visual and memory search (Cunningham & Wolfe, 2014; Shiffrin & Schneider, 1977; Wolfe, 2012), change detection (Droll et al., 2007; Rensink, 2002), and priming (Woltz & Was, 2007), among others. For any of these tasks, if some or all stimuli are repeated across the experiment, there is the possibility that reaction times might be inflated if the probed stimulus was previously seen. This could result in altering the magnitude of certain RT effects, and/or inflating RTs beyond what would be expected (e.g., longer search times). In addition, this effect may be even more pronounced for older adults (Oberauer, 2001). More research is required to determine whether the inflation of RTs for repeated stimuli with activated LTM representations generalises to other tasks, or whether this finding is simply a consequence of Sternberg-style memory tasks.

### Is activated LTM a continuous or discrete state?

If activated LTM is discrete (i.e., a representation is either active or not), then the behavioural consequences of the activated state (i.e., the intrusion effect) should be constant such that repeat and non-repeat objects produce intrusion effects of the same magnitude. Yet in the present study, we found that repetition increased the magnitude of the intrusion effect, lending support to the conclusion that activated LTM is continuous. Was it reasonable for us to question whether activated LTM *could* be discrete? Contemporarily, embedded-processes models of memory are described as having four levels of representation: LTM, activated LTM, the region of direct access, and the focus of attention (Oberauer, 2002; Oberauer & Hein, 2012). In support of activated LTM having the potential to be discrete, most models describe these levels as dichotomous, “all-or-none” states (Cowan, 1988, 2005; Cowan & Rachev, 2018; Oberauer, 2002). In addition, one of the consequences of changing the state of a memory representation is to alter the availability of that representation to conscious awareness (Cowan & Rachev, 2018). Importantly, access to consciousness appears to be dichotomous. For example, when participants rate the visibility of targets in an attentional blink paradigm on a continuous scale, they rarely use central values on the scale and instead report targets as being either fully visible or fully not visible (Sergent & Dehaene, 2004; Sy et al., 2021). Given that changing the memory state has a discrete effect on consciousness, one might have expected the consequences of activated LTM (on the intrusion effect) to also be discrete. Yet, our results favour the conclusion that activated LTM is continuous, suggesting that descriptions of embedded-processes models of memory may need to be updated. There are, however, some limits to the present results. Specifically, the observed effect of repetition on the intrusion effect can be explained without precluding activated LTM as a discrete state. For example, it could be the case that repetition increases the probability that an item is activated. In addition, it could be the case that activated LTM representations decay over time, and repetition slows the decay process. Finally, it could be the case that repetition enhances how precisely the object is represented, making it more likely that irrelevant probes successfully evoke a familiarity signal (Oberauer, 2002). Thus, while we interpret the current results as supporting continuous activated LTM, more research is needed to determine whether repetition only appears to enhance the intrusion effect.

### Summary

In the present study, we found that repeatedly encoding a set of visual objects into memory enhanced the consequences of activated LTM (i.e., the intrusion effect) by about 35%. This finding adds to the evidence that activated LTM is a continuous state, such that repetition can cause representations to be more activated; however, further research is needed to strengthen this conclusion. This finding also has implications for ongoing investigations of the role of activated LTM in supporting interactions between LTM and visual attention. Given that repetition enhances the effects of activated LTM, attentional processes that draw from activated LTM should be similarly affected by repetition. Moreover, researchers who study the interaction of attention and memory should consider repeating their stimuli to potentially enhance activated LTM’s ability to interface with perception, thereby increasing the size of their effect of interest.
